# Galantamine potentiates the neuroprotective effect of memantine against NMDA-induced excitotoxicity

**DOI:** 10.1002/brb3.118

**Published:** 2013-01-11

**Authors:** João P Lopes, Glauco Tarozzo, Angelo Reggiani, Daniele Piomelli, Andrea Cavalli

**Affiliations:** 1 D3 – Drug Discovery and Development Department, Istituto Italiano di TecnologiaVia Morego, 16163, Genova, Italy; 2 Departments of Anatomy and Neurobiology and Biological Chemistry, University of CaliforniaIrvine, CA, 92697-4621; 3 Department of Pharmacy and Biotechnologies, Alma Mater Studiorum, Bologna UniversityVia Belmeloro, 40126, Bologna, Italy

**Keywords:** Alzheimer's disease, drug combination, NMDA neurotoxicity, NR2B, polypharmacology, primary cortical neurons

## Abstract

The combination of memantine, an *N*-methyl-d-aspartate (NMDA) receptor antagonist, with an acetylcholinesterase inhibitor (AChEI) is the current standard of care in Alzheimer's disease (AD). Galantamine, an AChEI currently marketed for the treatment of AD, exerts memory-enhancing and neuroprotective effects via activation of nicotinic acetylcholine receptors (nAChRs). Here, we investigated the neuroprotective properties of galantamine in primary cultures of rat cortical neurons when given alone or in combination with memantine. In agreement with previous findings, we found that memantine was fully effective in reversing NMDA toxicity at concentrations of 2.5 and 5 μmol/L. Galantamine also completely reversed NMDA toxicity at a concentration of 5 μmol/L. The α7 and α4β2 nAChR antagonists, methyllycaconitine, and dihydro-β-erythroidine blocked the neuroprotective effect of galantamine, demonstrating the involvement of nAChRs. The combination of memantine with galantamine produced synergistic actions, such that full neuroprotective efficacy, was obtained at inactive concentrations of memantine (0.1 μmol/L) and galantamine (1 μmol/L). A similar potentiation was also observed when memantine was replaced with ifenprodil, suggesting a possible involvement of the NR2B subunit of the NMDA receptor. In summary, our study reports for the first time at a cellular level that memantine and galantamine interact on the same excitotoxic cascade and that the combination of these two drugs can result in a remarkable neuroprotective effect.

## Introduction

Alzheimer's disease (AD) is a progressive, irreversible, brain disease that destroys memory and thinking skills. According to a recent estimate, over 37 million people worldwide suffer from AD with a clear trend of growth in future due to the increase in the average age of the population (Alzheimer's Disease International's 2009). Clinically, AD is characterized by a behavioral decline of cognitive functions. Despite intensive research efforts, very few drugs are currently approved specifically for AD, which remains the largest unmet medical need in neurology (Citron 2010).

The current standard of care for advanced AD consists of combinations of memantine and an acetylcholinesterase (AChE; EC 3.1.1.7) inhibitor (AChEIs) such as donepezil, rivastigmine, or galantamine. Memantine was originally described as a low-affinity voltage-dependent noncompetitive antagonist of the glutamate ionotropic receptor subtype *N*-methyl-d-aspartate (NMDA) (Chen et al. 1992). Overactivation of the NMDA receptor (NMDAR) is involved in glutamate toxicity and neuronal death, which has been reported in different neurodegenerative diseases, including AD (Chen et al. 1992; Chen and Lipton 1997). Therefore, NMDAR antagonists in AD are expected to block neurotoxicity, thus sparing functional neurons and slowing down the loss of cognitive functions (Chen and Lipton 2006). Interestingly, because of its favorable tolerability profile, memantine is the only NMDAR antagonist currently approved for human use. Indeed, memantine shows a different pharmacological profile compared with other NMDAR antagonists (Lipton 2006). This is shown by its lack of typical NMDAR antagonist side effects coupled to some paradoxical findings, such as reversion of memory deficits in aged rats (Pieta Dias et al. 2007), enhancement of spatial memory in healthy animals (Minkeviciene et al. 2008), and improvement of cognitive and behavioral performance in man (Gauthier et al. 2005; Peskind et al. 2006; Schulz et al. 2011). Recent studies have reported that memantine acts preferentially on extrasynaptic NMDARs, which are thought to be linked to the neurotoxic effects of glutamate, while leaving untouched glutamate-mediated synaptic activity (Xia et al. 2010).

AChEIs are used in AD to counteract/delay cognitive decline. It is well established that cognitive decline in AD correlates with deficits in cholinergic function due to reduction of acetylcholine (ACh) levels (Davies and Maloney 1976; White et al. 1977). AChEIs preserve ACh from degradation, thus sustaining cholinergic neurotransmission. Galantamine is an AChEI currently marketed for the treatment of AD. Relevant to this study, in addition to its cognitive-enhancing effects, galantamine has also been reported to have neuroprotective activity against glutamate toxicity in rat neurons, possibly via stimulation of nicotinic ACh receptors (nAChRs) (Takada et al. 2003; Akasofu et al. 2006).

Herein, to achieve a better understanding of the neuroprotective profile of the galantamine/memantine combination, we studied the effect of these drugs, administered either separately or together, against NMDA-induced neurotoxicity in rat cortical neurons. We show that galantamine and memantine (or ifenprodil) are neuroprotective when given separately, as previously reported. Moreover, combinations of subactive concentrations of galantamine with memantine (or ifenprodil) can afford a full neuroprotective effect, suggesting a reciprocal potentiation in counteracting the excitotoxic cascade triggered by NMDA.

## Material and Methods

### Reagents

Neurobasal (NB) medium, B27 supplement, penicillin/streptomycin, l-glutamine, and fetal bovine serum (FBS) were from Gibco (Paisley, U.K.). Cytotoxicity detection (LDH, lactate dehydrogenase) and cell proliferation (MTT, 3-[4,5-dimethylthiazol-2-yl]-2,5-diphenyltetrazolium bromide) assay kits were acquired from Roche (Mannheim, Germany). Poly-d-lysine-coated plates were purchased from BD Biosciences (Bedford, MA). Memantine hydrochloride, ifenprodil hemitartarate, methyllycaconitine (MCC) citrate, dihydro-β-erythroidine (DHBE) hydrobromide, and AR-R17779 (ARR) hydrochloride were obtained from Tocris (Bristol, U.K.). *N*-Methyl-d-Aspartate, galantamine hydrobromide, MK-801, and all other reagents were from Sigma (Saint Louis, MO).

### Animals

Pregnant Sprague-Dawley female rats were obtained from Charles River Italia (Calco, Italy). The animals were maintained in a temperature- and humidity-controlled colony room under a 12-h day–night cycle and were individually housed in plastic cages, having free access to food and water ad libitum. All procedures were performed in compliance with Italian regulations on the protection of animals used for experimental and other scientific purposes (D.M. 116192), and with European Economic Community regulations (O.J. of E.C. L 358/1 12/18/1986).

### Cortical neuron isolation

Rat primary neuron cell cultures were obtained using previously described procedures by Agostinho and Oliveira (2003) with some modifications. In brief, the neocortices of 17-day embryos from Sprague-Dawley rats were collected and placed in a Ca^2+^ and Mg^2+^-free Krebs buffer. Following a trypsinization step, the cortices were mechanically dissociated and the Krebs buffer was replaced with NB medium supplemented with 2 mmol/L l-glutamine, penicillin (100 U/mL), streptomycin (100 U/mL), and 10% FBS. Cell counting was performed using a Nucleocounter NC-100 (Chemometec, Allerod, Denmark), and neurons were plated into 24-well poly-d-lysine-coated plates at a density of 0.25 × 10^6^ cells per well. The cultures were maintained at 37°C in a humidified atmosphere with 5% CO_2_/95% air. After 24 h in culture, the FBS-containing medium was replaced with NB supplemented with 2% B27. Ninety-six hours following the isolation, 10 μmol/L cytosine-d-arabinofuranoside was added to the medium. After 48 h, this medium was completely substituted with fresh NB/B27 medium and partial medium changes were performed on alternate days until the 13th day in vitro.

### Excitotoxicity

Excitotoxicity was induced by a 3-h exposure of the neuronal cultures to 100 μmol/L of NMDA, carried out at 37°C in a 4-(2-hydroxyethyl)-1-piperazineethanesulfonic acid (HEPES)-buffered solution containing 120 mmol/L NaCl, 5.4 mmol/L KCl, 0.8 mmol/L MgCl_2_, 20 mmol/L HEPES, 15 mmol/L glucose, and 0.01 mmol/L glycine.

### Cell treatments

**Figure 1:**
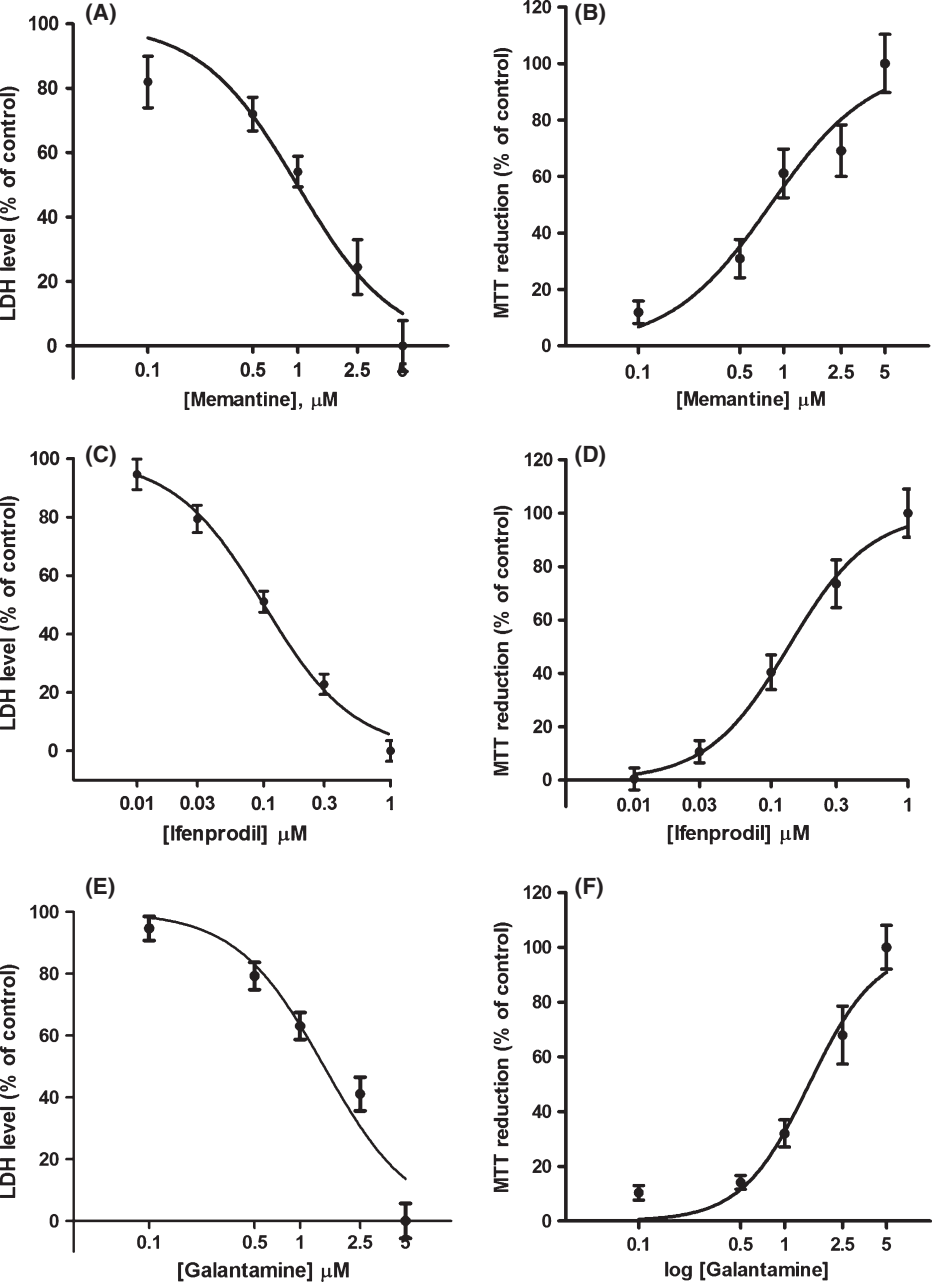
Protective effects afforded by individual treatments with memantine, ifenprodil, and galantamine against NMDA neurotoxicity in cultured rat cortical neurons. Memantine (A, B), ifenprodil (C, D), and galantamine (E, F) caused a significant and dose-dependent decrease in the toxicity of cortical neuron cultures when administered simultaneously to 100 μmol/L NMDA. Treatment duration was 3 h. Neurotoxicity was assessed using the MTT (A, C, E) or LDH (B, D, F) assays; *n* = 4 (A, B, C, D); *n* = 5 (E, F). NMDA, *N*-methyl-d-aspartate; MTT, 3-[4,5-dimethylthiazol-2-yl]-2,5-diphenyltetrazolium bromide; LDH, lactate dehydrogenase.

**Figure 2:**
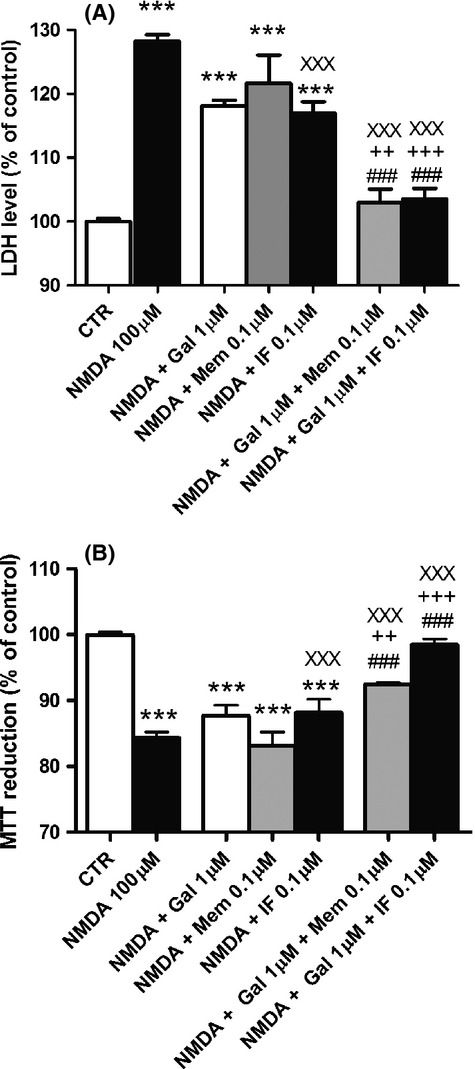
Protective effects afforded by combined treatments with memantine, ifenprodil, and galantamine against NMDA neurotoxicity in cultured rat cortical neurons. Treatment with galantamine (1 μmol/L)/memantine (0.1 μmol/L) or galantamine (1 μmol/L)/ifenprodil (0.1 μmol/L) combinations yielded significant neuroprotection against the toxicity of NMDA. Treatment duration was 3 h. Neurotoxicity was assessed using the LDH assay (A) or the MTT assay (B). ++: *P* < 0.01, +++: *P* < 0.001 compared with NMDA + Mem 0.1 μmol/L or IF 0.1 μmol/L; xxx: *P* < 0.001 compared with NMDA + Gal 1 μmol/L; ###: compared with NMDA, *n* = 4. NMDA, *N*-methyl-d-aspartate; MTT, 3-[4,5-dimethylthiazol-2-yl]-2,5-diphenyltetrazolium bromide; LDH, lactate dehydrogenase; IF, ifenprodil.

**Figure 3:**
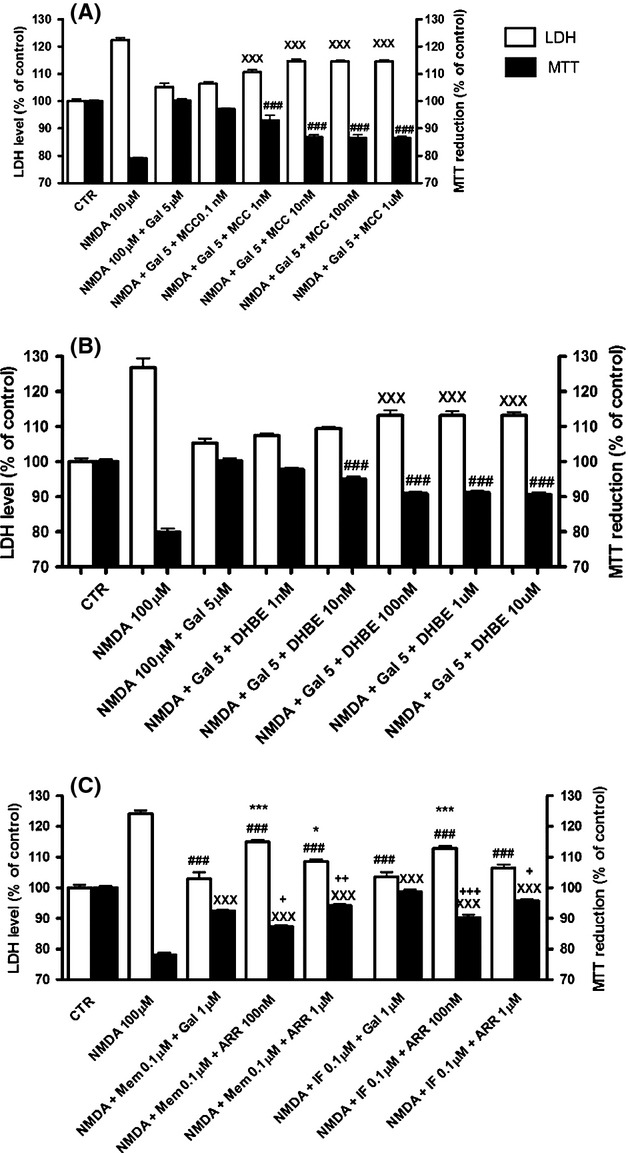
Blockade of α7 or α4β2 nAChRs decreases galantamine neuroprotection against NMDA toxicity, and activation of α7 nAChR with memantine or ifenprodil shows neuroprotective effect. Exposure of neuronal cultures to different concentrations of (A) methyllycaconitine (MCC) or (B) dihydro-β-erythroidine (DHBE) together with 5 μmol/L of galantamine and 100 μmol/L of NMDA resulted in a dose-dependent decrease in the neuroprotective effect of galantamine. Treatment duration was 3 h. Neurotoxicity was assessed using the LDH (white bars) or MTT (black bars) assays. ###, +++: *P* < 0.001 compared with NMDA + Gal 5 μmol/L, *n* = 3. (C) Administration of 0.1 and 1 μmol/L of AR-R17779 in combination with nonactive doses of memantine (0.1 μmol/L) or ifenprodil (0.1 μmol/L) prevents the neurotoxic effect of NMDA exposure in primary cultures of rat cortical neurons. Treatment duration was 3 h. Neurotoxicity was assessed using the MTT (black bars) or LDH (white bars) assays. ###: *P* < 0.001 compared with NMDA; *P < 0.05, ***P < 0.001 compared with NMDA + Mem 0.1 μmol/L + Gal 1 μmol/L or NMDA + IF 0.1 μmol/L + Gal 1 μmol/L, *n* = 3. NMDA, *N*-methyl-d-aspartate; nAChR, nicotinic acetylcholine receptor; MTT, 3-[4,5-dimethylthiazol-2-yl]-2,5-diphenyltetrazolium bromide; LDH, lactate dehydrogenase; IF, ifenprodil.

**Figure 4:**
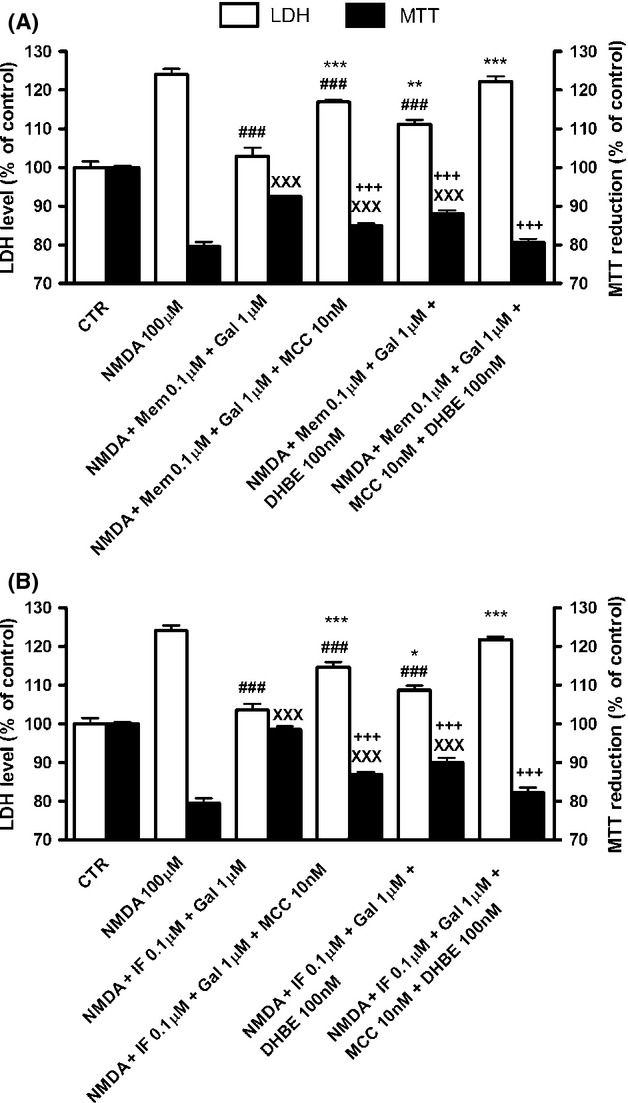
Combined administration of α7 and α4β2 nAChR antagonists abolishes the neuroprotective effect of memantine or ifenprodil plus galantamine. The neuroprotective effect of (A) memantine/galantamine and (B) ifenprodil/galantamine combinations was partially lost through treatment with either methyllycaconitine (MCC) or dihydro-β-erythroidine (DHBE). Simultaneous exposure to both nAChR antagonists completely blocks the protection of the NMDA antagonist/galantamine combination against 100 μmol/L of NMDA. Treatment duration was 3 h. Neurotoxicity was assessed using the MTT (black bars) or LDH (white bars) assays. ###: *P* < 0.001 compared with NMDA; **P < 0.01, ***P < 0.001 compared with NMDA + Mem 0.1 μmol/L + Gal 1 μmol/L or NMDA + IF 0.1 μmol/L + Gal 1 μmol/L, *n* = 3. NMDA, *N*-methyl-d-aspartate; nAChR, nicotinic acetylcholine receptor; MTT, 3-[4,5-dimethylthiazol-2-yl]-2,5-diphenyltetrazolium bromide; LDH, lactate dehydrogenase; IF, ifenprodil.

After 14 days in vitro, neurons were exposed to appropriate concentrations (see Figs. 1–4) of NMDA alone or in coadministration with memantine, ifenprodil, MK-801, galantamine, MCC citrate, DHBE hydrobromide, and ARR hydrochloride, separately or in different combinations. The duration of the treatment was 3 h.

### Neurotoxicity assessment

Neurotoxic damage was evaluated using the MTT and LDH assays according to the protocols provided by manufacturers.

#### MTT

MTT is reduced to formazan by metabolic active cells, and therefore, this conversion is directly related to the amount of viable cells. Briefly, after the cell treatments, MTT in a concentration of 5 mg/mL was added to the neuronal culture medium for 4 h at 37°C. After this incubation, a solubilization solution (10% sodium dodecyl sulfate [SDS] in 0.01 mol/L HCl) was added to the wells and left overnight at 37°C to dissolve the formazan crystals formed. Absorbance was measured at 570 nm on a Tecan Infinite M200 (Tecan, Männedorf, Switzerland) plate reader. Results were expressed as a percentage of the absorbance in control cells.

#### LDH

LDH is a cytosolic enzyme released to the medium when there is a rupture of the cell membrane. Therefore, the amount of LDH measured in the culture medium correlates to the number of dead cells.

Cell medium was collected and placed in a 96-well plate. A reaction mixture was then added and, after 30 min at room temperature in the dark, absorbance was measured at 490 nm.

### Statistical analysis

Results are expressed as means ± SEM. Statistical analysis was performed with Graphpad Prism software. Statistical significance was determined using an analysis of variance (ANOVA), followed by Dunnet's post hoc test. A two-tailed Student's *t*-test was used to compare different treatment conditions.

## Results

### NMDA toxicity is prevented by memantine, ifenprodil, and galantamine: single administration versus combination studies

Rat cortical neuronal cultures were exposed to concentrations of 50, 100, and 300 μmol/L of NMDA for 3 h. NMDA caused a dose-dependent increase of extracellular levels of LDH (increase of cell death), as well as a dose-dependent decrease of MTT (decrease of cell viability) (see Fig. S1).

As expected, the NMDA channel blocker MK-801 prevented NMDA toxicity, with IC_50_ values of 0.11 and 0.07 μmol/L, using the MTT and the LDH assays, respectively (see Fig. S1). Memantine also prevented the neurotoxic effect of NMDA in a dose-dependent manner (Fig. 1A) at concentrations between 0.1 and 5 μmol/L. IC_50_ values for memantine were 0.81 μmol/L (MTT) and 0.99 μmol/L (LDH). All IC_50_ values are reported in Table 1. It has been suggested that memantine might selectively interfere with extrasynaptic NMDARs (Xia et al. 2010). This subclass of receptors is highly enriched in the NR2B subunit (Thomas et al. 2006). Therefore, we tested ifenprodil, a selective antagonist of NR2B-containing NMDARs (Williams 1993), in the same experimental conditions. As shown in Figure 1B, ifenprodil exerted a protective effect against NMDA-mediated toxicity at concentrations between 0.01 and 1 μmol/L. IC_50_ values for ifenprodil were 0.13 μmol/L (MTT) and 0.1 μmol/L (LDH). As shown in Figure 1C, galantamine also produced a concentration-dependent neuroprotective effect, which was maximal at 2.5 μmol/L (MTT) and 5 μmol/L (LDH). IC_50_ values for galantamine were 1.48 μmol/L (MTT) and 1.44 μmol/L (LDH).

**Table 1:** IC_50_ obtained for memantine, ifenprodil, and galantamine on NMDA-induced neuronal cell death protection in the two different assays, MTT and LDH

	MTT metabolism		LDH levels	
		
	IC_50_ μmol/L (95% CI; *n*)	Hill slope	IC_50_ μmol/L (95% CI; *n*)	Hill slope
Memantine	0.82 (0.58–1.14; 3)	1.2	0.99 (0.75–1.31; 4)	−1.3
Ifenprodil	0.13 (0.10–0.18; 3)	1.5	0.10 (0.08–0.12; 5)	−1.2
Galantamine	1.48 (1.19–1.83; 3)	1.8	1.44 (1.19–1.75; 5)	−1.5

NMDA, *N*-methyl-d-aspartate; MTT, 3-[4,5-dimethylthiazol-2-yl]-2,5-diphenyltetrazolium bromide; LDH, lactate dehydrogenase. IC_50_ values were calculated by nonlinear regression analysis of log[concentration]/inhibition curves using GraphPad Prism 5 (GraphPad Software Inc., CA) applying a variable slope curve fitting using five different concentrations in quadruplicate and are expressed as means with corresponding 95% confidence interval (CI) from 3 to 5 independent experiments.

Next, we evaluated the combination of galantamine with memantine or ifenprodil. As shown in Figure 2, ineffective concentrations of galantamine (1 μmol/L) and memantine (0.1 μmol/L) were fully neuroprotective when administered in combination. This points to a possible reciprocal potentiation mechanism. In the same experimental conditions, we also tested the combination of ineffective concentrations of galantamine and ifenprodil. In this case too, we found that the galantamine/ifenprodil combination showed a remarkable neuroprotective effect (Fig. 2), much higher than that observed when the two compounds were administered separately.

### Galantamine exerts its neuroprotective effect via α7 and α4β2 nicotinic acetylcholine receptors

Galantamine does not bind to NMDARs (Simoni et al. 2012). Therefore, the reported effect against the NMDA-induced toxicity cannot be ascribed to the inhibition of these receptors. Galantamine increases ACh concentration *via* inhibition of AChE. In addition, it has been reported that galantamine is an allosteric modulator of nAChRs (Maelicke and Albuquerque 2000; Samochocki et al. 2003). Therefore, we assessed the role of nAChRs by blocking the α7 and α4β2 nAChRs, which are the most affected nAChRs subtypes in AD. Administration of MCC, an α7 nAChR antagonist, partially blocked the neuroprotective effect of galantamine (5 μmol/L) in a concentration-dependent manner, reaching a maximal effect at 10 nmol/L (Fig. 3A). Similarly, DHBE, an α4β2 nAChR antagonist, attenuated the protective effect of galantamine, although to a lesser extent than did MCC (Fig. 3B). To further test the possible role of α7 nAChR, we evaluated the effect of the α7 agonist ARR in potentiating the neuroprotective effect of memantine or ifenprodil (Fig. 3C). Our data show that ARR potentiated the effect of both memantine and ifenprodil, although to a lesser extent when compared with galantamine.

Finally, we treated cells with the memantine/galantamine combination and then with MCC and/or DHBE. Our results revealed a decreased potentiating effect of galantamine with either MCC or DHBE (Fig. 4A). When the two compounds were given simultaneously, the protective effect of the memantine/galantamine combination was completely lost. These experiments were repeated with the ifenprodil/galantamine combination, obtaining similar results (Fig. 4B).

## Discussion

Overactivation of NMDARs leads to neuronal death in different neurodegenerative conditions, including AD (Chen and Lipton 2006). Our results confirm previous data indicating that memantine prevents NMDA-induced excitotoxicity in rat neuronal cultures (Chen et al. 1992; Volbracht et al. 2006). Recent studies have suggested that memantine could preferentially block the extrasynaptic NMDARs, leaving untouched the synaptic receptors (Xia et al. 2010). It has been reported that extrasynaptic NMDARs are enriched of NR2B subunits (Thomas et al. 2006). Therefore, we tested ifenprodil, a selective antagonist of NR2B-containing NMDARs (Williams 1993), and showed that this compound was also able to block NMDA toxicity at a concentration approximately 10-fold lower than that of memantine.

It has been previously reported that galantamine exerts neuroprotective effects in rat cortical neurons exposed to β-amyloid (Kihara et al. 2004; Melo et al. 2009) or to glutamate (Takada et al. 2003). Galantamine also halts in vivo apoptosis in ischemic rat brains (Lorrio et al. 2007).

In this study, we have shown that galantamine was effective against NMDA-induced death in primary rat cortical neurons by a mechanism involving α7 and α4β2 nAChRs, in agreement with previously published results (Takada-Takatori et al. 2006). It is noteworthy that galantamine has been shown to selectively potentiate NMDA receptor activity (Moriguchi et al. 2004). Conversely, in a combined treatment with the two drugs, memantine was able to block tonic NMDA currents and Ca^2+^ influx promoted by galantamine, seemingly acting on the extrasynaptic NMDA channels, while synaptic NMDA currents were spared (Zhao et al. 2006). Therefore, the combined treatment should prevent the extrasynaptic NMDA overexcitation while promoting synaptic glutamatergic signaling in patients. When we evaluated the effect of the memantine/galantamine combination against NMDA-induced neurotoxicity, we observed a substantial increase of potency with respect to each compound administered separately, suggesting a reciprocal potentiation. This effect was replicated when memantine was replaced with ifenprodil, a selective antagonist of NR2B-containing NMDARs.

The mechanism by which memantine (or ifenprodil) and galantamine potentiate each other's efficacy in the NMDA-induced rat cortical neuronal death is yet to be elucidated. Our findings are consistent with the hypothesis that extrasynaptic NMDA receptors (i.e., N2RB-enriched NMDA receptors) and nAChRs are the molecular targets for such a potentiation. Both receptors are likely to be present on the same cellular neurotoxic path, and both nAChRs activation (Akaike et al. 2010) and NR2B blockade (Liu et al. 2007) lead to the increase of phosphorylated Akt levels and prevention of neuronal death. This could therefore account for the fact that galantamine can turn off NMDA-mediated neurotoxicity and, most importantly, that the simultaneous administration of galantamine and an NMDAR antagonist can provide a significantly greater inhibition of the neurotoxic path, as compared with each single compound given separately. We hypothesize that galantamine and memantine can tackle neurotoxicity at two different levels within the same cascade. Despite the limitations of the present cell-based experiments, our findings could also help to elucidate the potentiation observed in AD patients treated with a combination of the two drugs (Grossberg et al. 2006).

In conclusion, our results support the view that in disease conditions such as AD, the simultaneous administration of galantamine and NR2B-containing NMDAR antagonists (or extrasynaptic NMDA antagonists) can attenuate the neuronal loss caused by excessive NMDAR activation. In this context, this study lays the foundation for the design of dual AChE/NMDAR-NR2B inhibitors (Simoni et al. 2012), which could offer a novel therapeutic option for the treatment of AD.

## Supporting Information

**Figure S1.** Effect of NMDA in the viability of primary neuronal cultures and neuroprotection afforded by MK-801.
