# Supplementary figures and images for: Galantamine potentiates the neuroprotective effect of memantine against NMDA-induced excitotoxicity
Source: Brain Behav. 2013 Jan 11;3(2):67–74. doi: 10.1002/brb3.118 (PMC3607148; doi:10.1002/brb3.118)

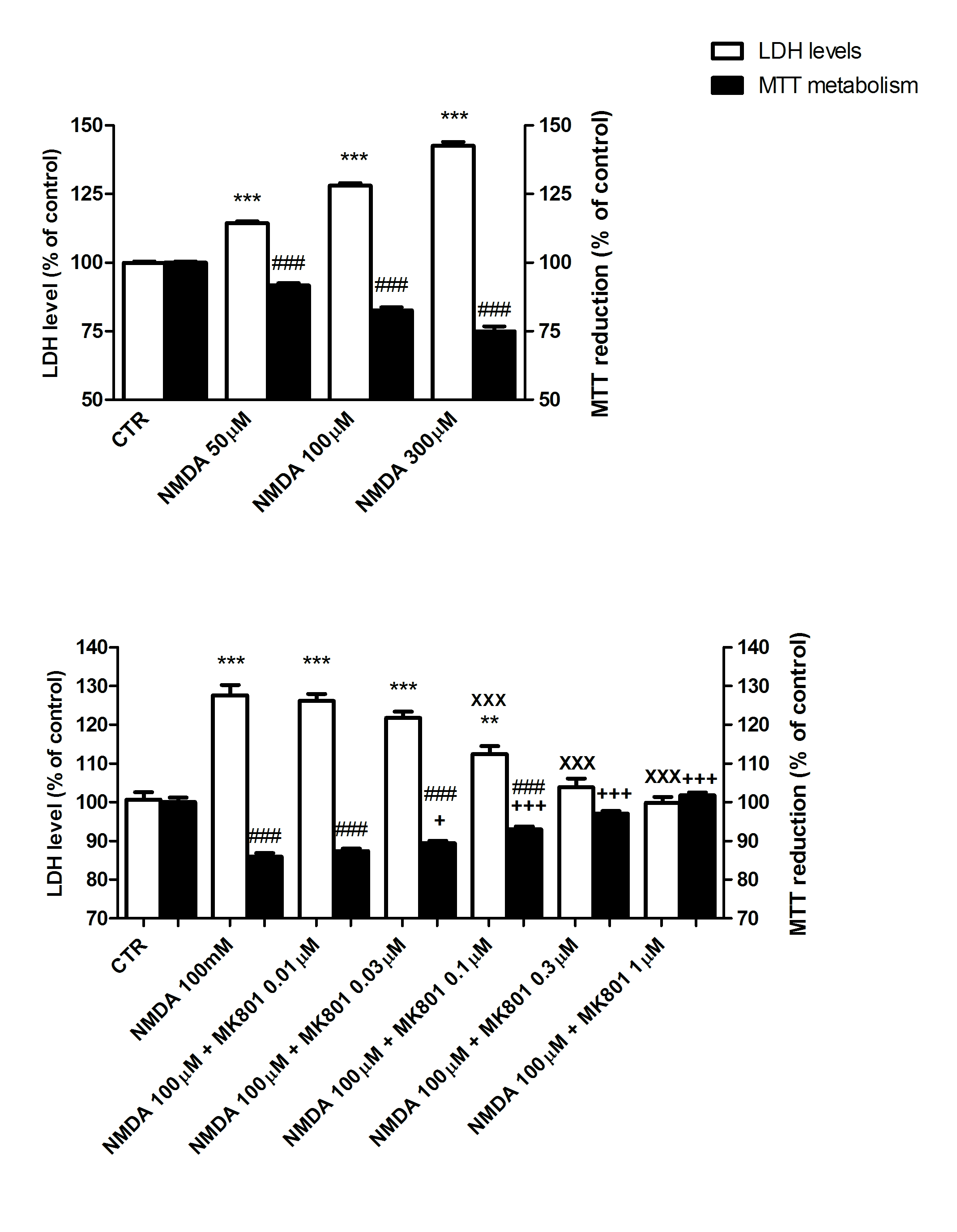

Supplement: Supplementary file 1 [file brb30003-0067-SD1.tif]
